# Varying Spectrum of Frosted Branch Angiitis: A Curated Constellation of Cases

**DOI:** 10.1155/crop/8844545

**Published:** 2025-02-10

**Authors:** Avik Dey Sarkar, V. Muthukrishnan, Naresh Babu Kannan, Ananya Goswami

**Affiliations:** Department of Vitreoretinal Services, Aravind Eye Hospital, Madurai, India

**Keywords:** flecked retina, frosted branch angiitis, progressive outer retinal necrosis, trauma, tuberculosis

## Abstract

**Purpose:** The purpose of this study is to elucidate varying etiology and presentations of frosted branch angiitis (FBA), a rare immune-mediated retinal vasculitis.

**Methods:** In this case series, four curated cases confined to varying spectrums of FBA have been described. Detailed fundoscopic documentation with pertinent imaging was performed in all the cases. The cases were managed adequately as per protocol and retrospectively analyzed.

**Results:** Our first case is unique as it has been rarely reported in extant ophthalmic literature. The case is presented as progressive outer retinal necrosis in cytomegalovirus (CMV) retinitis accompanied by FBA. The second case is a Kleiner Type 3 FBA with viral prodrome. Our third case is the fourth reported case in literature of FBA following penetrating trauma. This case is instrumental in confirming the hypothesis of Type 3 hypersensitivity reaction in the slow deposition of immune complexes in the periarteriolar region as a pathophysiology for FBA. The fourth case is the first reported case of coexisting bilateral flecked retina and FBA. It also had an unusual association with tuberculosis mimicking tubercular vasculitis.

**Conclusion:** This gamut of unique cases may contribute to a better understanding of the variability of clinical presentation of FBA in a more detailed manner for future reference.

## 1. Introduction

Frosted branch angiitis (FBA) is a rare immune-mediated retinal vasculitis that presents with translucent widespread perivascular exudate resembling frosted branches of a tree [1]. The entity was first described in ophthalmic literature by Ito et al. in 1976 in a 6-year-old child [2]. Due to its varied etiologic presentations, Kleiner et al. classified FBA into three types based on their origin [3]. Type 1 was described as a paraneoplastic syndrome in leukemia or lymphoma and is caused by direct vascular invasion of cancer cells. Type 2 was termed as the secondary variety, characterized by immune complex deposition following infective or immune prodrome. Type 3 was described as an idiopathic variant. To date, at least 240 cases of FBA have been documented in ophthalmic literature [4].

This article meticulously presents four curated cases of FBA with rare and novel features. One case had a novel coexistence of progressive outer retinal necrosis (PORN) with cytomegalovirus (CMV) retinitis, and one other had a rare presentation of FBA in the background of benign fleck retina. Ocular trauma followed by late presentation of FBA was reported in one patient. One intriguing case with Type 3 FBA is described in this series.

## 2. Case Reports

### 2.1. Case 1

This is a novel case description of FBA, reporting PORN in concurrent CMV retinitis.

#### 2.1.1. Presentation

A 69-year-old woman presented with sudden-onset decreased vision in the left eye (OS) for 1 week. She did not have any significant medical or ocular comorbidities. On detailed family history, she revealed that her husband was seropositive for human immunodeficiency virus (HIV) infection and was treated with antiretroviral therapy. Her presenting best corrected visual acuity (BCVA) was 20/60 on Snellen's chart. Intraocular pressure (IOP) was within normal limits, and anterior segment findings were unremarkable.

**Figure 1:**
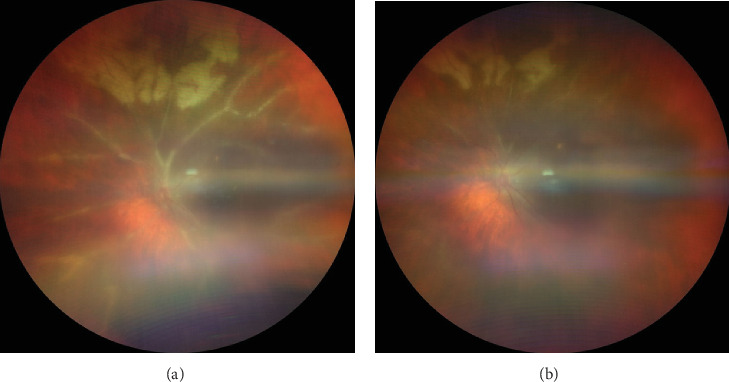
(a) OS fundus image showing frosted branch angiitis involving first-order vessels of all quadrants with fluffy perivascular exudation in the superior quadrant without any significant vitritis suggestive of progressive outer retinal necrosis with frosted branch angiitis. (b) OS fundus image of posttreatment phase showing resolution of perivascular sheathing from all quadrants except in the superior quadrant along with reduction in perivascular exudation.

On dilated fundoscopy, retinitis with perivenular sheathing was seen all over 360° and was most prominent superiorly. She also presented with a PORN-like clinical picture in the midperiphery with a mild degree of vitritis. Based on clinical findings, it was diagnosed as Type 2 FBA with PORN in the OS (Figure 1(a)).

#### 2.1.2. Investigation

The patient was investigated further at an integrated counselling and testing center (ICTC), and serology was found to be HIV positive. CD_4_ T cell levels dropped to 91 cells/*μ*L. The serum angiotensin-converting enzyme (ACE) levels and erythrocyte sedimentation rate (ESR) were elevated (Figure 1(b)).

#### 2.1.3. Treatment

Antiretroviral therapy was initiated. At 1-month follow-up, the patient underwent serological marker assessment for CMV antibodies, vitreous biopsy sampling, and intravitreal injection of ganciclovir in the OS. Her anti-CMV immunoglobulin M (IgM) and anti-CMV immunoglobulin G (IgG) titres were elevated on serological testing, and she tested positive for CMV DNA in vitreous biopsy when polymerase chain reaction (PCR) testing was performed. She received a second dose of intravitreal ganciclovir in the same eye, and oral valganciclovir was administered.

#### 2.1.4. Outcome

On 6-month follow-up, the perivenular sheathing had resolved except for the superior quadrant and her BCVA improved to 20/40 on Snellen's chart. Oral valganciclovir therapy was continued further.

### 2.2. Case 2

This is a unique case of FBA, where in spite of the patient having possible viral prodrome, a presumptive diagnosis of Type 3 FBA was concluded based on clinical evidence and investigational parameters.

#### 2.2.1. Presentation

A 14-year-old male child presented with sudden-onset decreased vision in both eyes (OU) for the last 3 days. According to his parents, he had a history of febrile episode 1 week prior to presentation. The systemic and family history was insignificant. The presenting BCVA was 20/120 in the right eye (OD) and 20/80 in the OS on Snellen's chart. On ophthalmoscopic evaluation, bilaterally dilated tortuous veins with perivenular sheathing and minimal intraretinal hemorrhage were noted in all quadrants.

#### 2.2.2. Investigation

Hematological parameters showed values within normal limits. Acid-fast smear of sputum, Mantoux test, and tuberculin skin test were negative. The specific antibody titres for herpes group of viruses, toxoplasma, mycoplasma, syphilis, and HIV were negative. Abdominal ultrasound screening, chest computed tomography, and brain magnetic resonance imaging were normal and did not show any signs of occult malignancy. Even after a thorough work-up, no concluding evidence regarding the etiology could be established other than preceding febrile episode, indicating transient viral illness.

#### 2.2.3. Treatment

**Figure 2:**
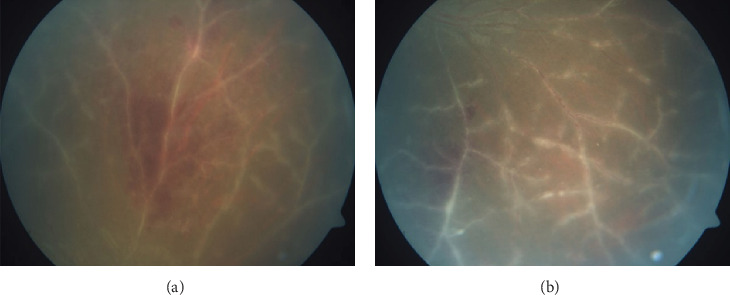
(a) Sectoral fundus image showing peripheral area in the OD with frosted branch–like appearance of vessels and diffuse intraretinal hemorrhage as seen usually in Type 3 Kleiner's variety of frosted branch angiitis. (b) OS fundus image showing posterior fundus with perivascular sheathing involving multiple vessels in all quadrants.

Hence, the case was diagnosed as Type 3 FBA and treated with empirical valaciclovir 1000 mg thrice daily and prednisolone 40 mg/day. The child was vigilantly monitored (Figures 2(a) and 2(b)).

In a couple of months, clinical improvement was observed and the child gained BCVA 20/30 in OU. Oral corticosteroid dose was gradually tapered. After 2 months, although BCVA remained stable in OU, peripheral exudation was noted bilaterally. Fundus fluorescein angiography (FFA) revealed peripheral areas of capillary nonperfusion (CNP) along with late leakage at the junction of vascular and avascular retina in OU. The child underwent bilateral FFA-guided targeted laser photocoagulation for the CNP areas.

#### 2.2.4. Outcome

On follow-up, FFA was repeated after 2 months and CNP areas were found to be stable in OU. The child was closely monitored over a period of 18 months, and no signs of recurrence were noted.

### 2.3. Case 3

This is the fifth case of FBA reported in ophthalmic literature with preceding history of significant ocular trauma.

#### 2.3.1. Presentation

**Figure 3:**
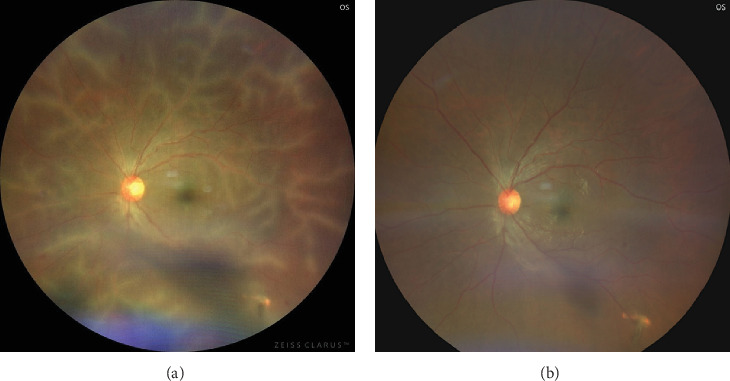
(a) Fundus image of OS posterior pole showing extensive perivascular exudation along the vessels in all quadrants on presentation. (b) After treatment, all the exudation has resolved and the posterior pole appears within normal limits.

In this interesting case scenario, a 30-year-old male presented with an open globe injury with full-thickness corneal tear and iridodialysis in the OS following a penetrating injury. He was otherwise a healthy normal individual and did not have any significant medical history. He underwent immediate primary repair of corneal tear and iridodialysis and was compliant with routine postoperative medications After 6 weeks of initial management, the patient presented with gradual painless diminution of vision in the OS. There was no febrile episode or other ailments during this period. The presenting BCVA was 20/200. Fundus examination revealed venous tortuosity with translucent retinal perivascular infiltration predominantly affecting venules from the posterior pole to the periphery in the OS (Figure 3(a)).

#### 2.3.2. Investigation

**Figure 4:**
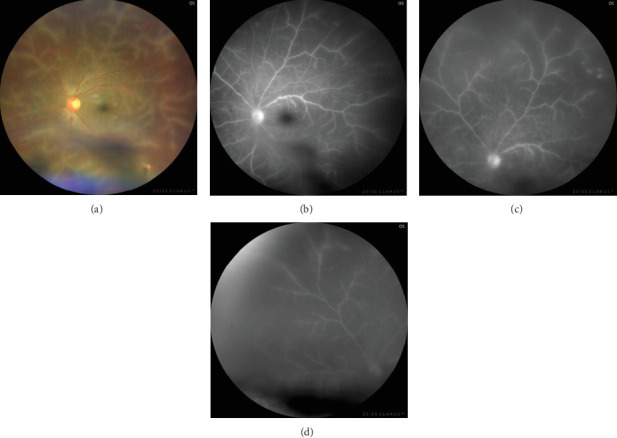
(a) Wide-angled fundus image of OS posterior pole showing extensive perivascular exudation along the vessels in all quadrants on presentation. (b–d) Wide-angled FFA showing perivascular hyperfluorescence indicating perivascular sheathing and widespread areas of peripheral capillary nonperfusion as hypofluorescent areas.

FFA showed hyperfluorescence along the venules in all quadrants with peripheral CNP areas without any signs of late leakage (Figures 4(a), 4(b), 4(c), and 4(d)). Optical coherence tomography (OCT) revealed normal foveal contour. The fellow eye was unaffected (Figure 3(b)). A battery of serological tests revealed all the titers to be within normal limits. Mantoux test and specific serological tests for syphilis and HIV were negative.

#### 2.3.3. Treatment

The patient was diagnosed with a case of Type 2 FBA, and oral prednisolone at a dose of 1 mg/kg/day dose was initiated. Topical steroids and cycloplegic were coadministered. After a fortnight of treatment, there was resolution of perivascular sheathing and BCVA in the OS improved to 20/80.

#### 2.3.4. Outcome

Complete resolution of all perivascular exudates were recorded in 6 weeks, and no signs of peripheral exudation noted. Gradual tapering of systemic steroid was done. The last follow-up visit at 7 months showed no signs of recurrence.

### 2.4. Case 4

This is a rare case with coexistence of FBA and benign fleck retina.

#### 2.4.1. Presentation

**Figure 5:**
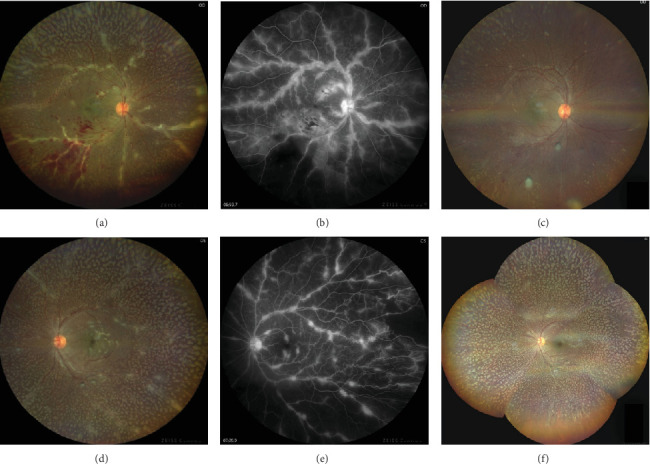
(a) Fundus image of the OD shows extensive, opaque, interrupted perivascular sheathing involving first-order vessels with superficial intraretinal hemorrhage involving inferotemporal quadrant suggestive of Type 2 Kleiner's variety of frosted branch angiitis. Another unique feature noted was the preretinal fluffy exudates over the posterior pole supporting intravitreal involvement of frosted branch angiitis in cases with excessive exudation. Along with this, benign flecks were noted all over the fundus. (b) Fundus image of the OS shows similar features as seen in the OD, although intraretinal hemorrhage was absent. Preretinal exudative clumps and benign flecks were also noted. (c) Fundus fluorescent image showing perivascular hyperfluorescence indicating leakage and exudation and blocked fluorescence over inferotemporal quadrant that corresponds to superficial intraretinal hemorrhage. (d) Similar to image (c). Fundus fluorescent angiography in the OS shows perivascular hyperfluorescence in all quadrants although the involvement is patchier and more discreet and a small area of temporal peripheral capillary nonperfusion. (e) Posttreatment fundus image of the OD showing resolution of frosted branched appearance with reduction in intraretinal hemorrhage but persistence of preretinal exudates. (f) Posttreatment montage fundus photography of the OS elaborates on 360° flecks over the retina with complete resolution of perivascular exudation and incomplete resolution of preretinal exudative clumps.

This last case is a unique one as a 21-year-old girl presented with sudden-onset reduced vision and photophobia in OU for 1 week. She did not have any preceding history of trauma or fever. Her medical and family history was unremarkable. She had a BCVA of 20/80 in the OD and 20/30 in the OS on presentation. On fundoscopy, she was diagnosed with a case of benign fleck retina and moderate vitreous haze was present. Splinter hemorrhages on the posterior pole and perivascular sheathing were observed in OU. It was also noted that the exudates not only were intraretinal but also involved the posterior hyaloid face (Figures 5(a) and 5(b)).

#### 2.4.2. Investigation

FFA showed contiguous, granular, and opaque perivascular sheathing in the OD, compared to the OS in which sheathing was more discrete and it has a beaded appearance (Figures 5(c) and 5(d)). The case was diagnosed as Type 2 FBA in a patient with a bilaterally flecked retina. The complete hemogram showed borderline elevated ESR, and Mantoux test turned out to be reactive. The other serological investigations and chest x-ray reports were within normal limits, and no systemic involvement of tuberculosis was recorded.

#### 2.4.3. Treatment

Treatment was initiated with antitubercular therapy (ATT), and oral prednisolone was administered in a dose of 1 mg/kg/day. On 1-month follow-up, BCVA improved to 20/20 in the OS, whereas it remained static in the OD. The perivascular sheathing was noted to be drastically reduced in OU. A dull foveal reflex prompted an OCT scan which revealed a massive cystoid macular edema (CME) with a central macular thickness of 595 *μ*. A posterior subtenon injection of 0.1 mL of triamcinolone acetonide (40 mg/mL) was administered. IOP was assessed on follow-up visits and was found to be normal.

#### 2.4.4. Outcome

On 6-week follow-up, there was complete resolution of CME and BCVA improved to 20/20 in OU. There was resolution of perivascular sheathing with absence of exudation. A tapering dose of oral steroid was added to the standard regimen of ATT. There was no recurrence till her last follow-up visit, which was 6 months since the initial presentation (Figures 5(e) and 5(f)).

The details of all the cases are given in Table 1 in a listed format. Informed consent from all the patients was obtained.

## 3. Discussion

In this case series, four curated cases confined to varying spectrum of FBA have been described. According to Kleiner's classification, our second patient belongs to Type 3 and the rest are Type 2. A bimodal peak of incidence of FBA in childhood and early adulthood has been described [5]. The patients reported in our case series conform to the aforementioned age groups barring the first case reported in an elderly female. Traditionally, FBA has been described as a bilateral asymmetric disease. A literature review of 240 cases reveals bilaterality in 132 patients (55%). In our series, bilaterality was observed in two out of four patients.

CMV infection has been reported as the leading cause for Type 2 FBA. Till date, at least 131 cases of Type 2 FBA have been described out of which 28.2% of cases had documented evidence of CMV infection [4]. None of the cases have mentioned any association of CMV retinitis with acute retinal necrosis (ARN) or PORN presenting with FBA [4]. There is an isolated report of HSV infection along with FBA that progressed to ARN despite prompt treatment with acyclovir [6]. Our first case is unique as it is the first report in literature that presented with PORN in CMV retinitis accompanied by FBA. There have been isolated case reports of CMV retinitis with FBA or PORN with FBA but never in coexistence [7]. In addition, this case was the oldest patient recorded in literature with Type 2 FBA associated with CMV infection. Viral invasion of blood vessel or hypersensitivity reaction following CMV infection has been speculated for occurrence of FBA in CMV infection. FBA has also been reported to present as immune recovery uveitis in cases with HIV-related CMV infection following initiation of highly active antiretroviral therapy (HAART) [8]. In our case, the patient presented with FBA before initiation of HAART. Another interesting aspect was the unilateral presentation despite an underlying systemic condition. According to existing literature, the deposition of the immune complex in the periarterial region depends not only on systemic immune status but also on the ocular immune profile and integrity of the blood vessels itself [8].

Our second reported case belongs to Kleiner's Type 3 FBA due to association with viral prodrome, no definite history of other systemic illness, and clinically conspicuous contiguous sheathing. Ito et al. described their first case as a contiguous florid periarterial sheathing with limited retinal hemorrhage [2]. It has been suggested that the clinical picture elucidated by Ito et al. inclined more towards Type 3 FBA [4].

The third case is posttraumatic Kleiner's Type 2 FBA of unilateral presentation. Only four cases of posttraumatic FBA have been described in literature [9–12]. FBA following penetrating trauma is a relatively rare phenomenon, and the presentation is delayed as noted in most of the cases reported. Arend and Thurau [11] and Annamalai and Biswas [12] in their published articles have noticed a lucid interval of 4 and 8 months, respectively, from the injury to the onset of FBA. This helps in hypothesizing the role of Type 3 hypersensitivity reaction in slow deposition of immune complexes in the periarteriolar region [13].

Our fourth case is unique as it is the first case of coexisting flecked retina and FBA. The possible association with tuberculosis makes the scenario more enigmatic. There have been reports regarding the association of tuberculosis and FBA with proven evidence of systemic tuberculosis [14–17]. One of the reported cases was finally documented as tubercular vasculitis rather than FBA [15]. By definition, tubercular vasculitis is more peripheral and usually spares the posterior pole vasculature. However, in our case, the first-order vessels emanating from the disc were significantly involved. Also, the involvement was patchy, irregular, granular, and opaque pointing towards Kleiner's Type 2 FBA.

## 4. Conclusion

The varied presentations of FBA are portrayed in this series. The unique features may help in better understanding of the entity and enrich existing literature on FBA for future reference.

## Figures and Tables

**Table 1:** Summary of all the cases presented with frosted branch angiitis in the article.

	**Age/gender**	**Systemic**	**Laterality**	**Clinical diagnosis**	**Kleiner's FBA type**	**Management**
Case 1	69/female	HIV	Unilateral	PORN	Type 2	Oral valaciclovir, intravitreal ganciclovir
Case 2	14/male	Nil	Bilateral	Possible viral fever	Type 3	Oral valaciclovir and prednisolone, FFA-guided PRP
Case 3	30/male	Nil	Unilateral	Open globe injury	Type 2	Oral prednisolone
Case 4	21/female	Nil	Bilateral	Flecked retinopathy, ocular TB	Type 2	ATT, oral prednisolone, PST for CME

## Data Availability

The article has restricted data under the control of the authors. That data can only be produced or published after due request to the authors only.
